# Septins localize to microtubules during nutritional limitation in *Saccharomyces cerevisiae*

**DOI:** 10.1186/1471-2121-9-55

**Published:** 2008-10-01

**Authors:** M Evangelina Pablo-Hernando, Yolanda Arnaiz-Pita, Hiroyuki Tachikawa, Francisco del Rey, Aaron M Neiman, Carlos R Vázquez de Aldana

**Affiliations:** 1 Instituto de Microbiología Bioquímica, Departamento de Microbiología y Genética, CSIC/Universidad de Salamanca, 37007, Salamanca, Spain; 2 Department of Applied Biological Science, Graduate School of Agricultural and Life Sciences, University of Tokyo Tokyo 113-8657, Japan; 3 Department of Biochemistry and Cell Biology, SUNY Stony Brook, Stony Brook, NY 11794-5215, USA

## Abstract

**Background:**

In *Saccharomyces cerevisiae*, nutrient limitation stimulates diploid cells to undergo DNA replication and meiosis, followed by the formation of four haploid spores. Septins are a family of proteins that assemble a ring structure at the mother-daughter neck during vegetative growth, where they control cytokinesis. In sporulating cells, the septin ring disassembles and septins relocalize to the prospore membrane.

**Results:**

Here, we demonstrate that nutrient limitation triggers a change in the localization of at least two vegetative septins (Cdc10 and Cdc11) from the bud neck to the microtubules. The association of Cdc10 and Cdc11 with microtubules persists into meiosis, and they are found associated with the meiotic spindle until the end of meiosis II. In addition, the meiosis-specific septin Spr28 displays similar behavior, suggesting that this is a common feature of septins. Septin association to microtubules is a consequence of the nutrient limitation signal, since it is also observed when haploid cells are incubated in sporulation medium and when haploid or diploid cells are grown in medium containing non-fermentable carbon sources. Moreover, during meiosis II, when the nascent prospore membrane is formed, septins moved from the microtubules to this membrane. Proper organization of the septins on the membrane requires the sporulation-specific septins Spr3 and Spr28.

**Conclusion:**

Nutrient limitation in *S. cerevisiae *triggers the sporulation process, but it also induces the disassembly of the septin bud neck ring and relocalization of the septin subunits to the nucleus. Septins remain associated with microtubules during the meiotic divisions and later, during spore morphogenesis, they are detected associated to the nascent prospore membranes surrounding each nuclear lobe. Septin association to microtubules also occurs during growth in non-fermentable carbon sources.

## Background

Meiosis is a specialized type of cell division that produces the haploid cells needed for sexual reproduction. Completion of meiosis is normally coupled to differentiation programs that produce gametes. In *Saccharomyces cerevisiae*, sporulation is triggered in diploid cells in the absence of nitrogen and in the presence of a non-fermentable carbon source, such as acetate [1]. During meiosis, one round of S phase is followed by two meiotic divisions that generate four haploid nuclei. During meiosis II, a differentiation program starts, resulting in the formation of spores (reviewed in [2]). Encapsulation of the four haploid nuclei into spores requires the modification of the spindle pole body (SPB) by the incorporation of several meiosis-specific proteins to form the meiosis II outer plaque [3-5]. Once the meiosis II SPB outer plaque has been assembled, secretory vesicles coalesce on this structure to form flattened double-membrane sheets termed prospore membranes [6-8]. The prospore membranes continue to expand during meiosis II, and at the time of nuclear division each prospore membrane completely engulfs the nuclear lobe to which it is anchored via the SPB. At the end of meiosis II, each prospore membrane closes around the daughter nucleus (and associated cytoplasm), creating immature spores. The final step of spore formation requires the *de novo *synthesis of a spore wall, which occurs in the lumen between the two prospore membranes (reviewed in [2]). This spore cell wall is responsible for the stress resistance of spores.

Septins are a family of filament-forming proteins that play crucial roles in morphogenesis. In yeasts, during vegetative growth septins are normally assembled in a ring-like structure at the site where the septum between mother and daughter cells is to be placed [9-12]. The structure, dynamics and regulation of septins are well known in *S. cerevisiae*. Their biological functions in vegetative yeast cells are two-fold [10,12-15]: first, they act as a scaffold for anchoring other regulatory proteins that control the establishment of the septation site and cytokinesis, bud-site selection, and spindle positioning. Second, they form a diffusion barrier to control the molecular trafficking between mother and daughter cells [16]. There are seven septin genes in *S. cerevisiae*. In vegetative cells, the septin ring is composed of five septins: Cdc3, Cdc10, Cdc11, Cdc12 and Shs1/Sep7. During the sporulation program, two additional septins, Spr3 and Spr28, are specifically induced [17-19]. In meiotic cells, transcription of two of the vegetative septins -*CDC3 *and *CDC10- *is also upregulated, while expression of *CDC11, CDC12 *and *SHS1 *is similar to vegetative cells [17,19-21]. In addition, the Ssp1 protein was recently proposed to be a non-conventional septin required for prospore membrane closure [22]. During spore formation, septins disappear from the bud neck and relocalize later to specific regions of the developing spore. In early meiosis II, septins are assembled as four ring-like structures surrounding each SPB, after which they expand into a pair of bars or sheets that run parallel to the long axis of the prospore membrane as it grows [23]. After closure of the prospore membrane, the septins become diffusely localized at the spore periphery [17]. Even though septins are closely associated with the prospore membrane, disruption studies have revealed only modest sporulation defects in septin mutants, suggesting that septins may function redundantly in spore formation [2,17,19]. Septin organization onto the prospore membrane requires Gip1, a sporulation-specific regulatory subunit of the Glc7 protein phosphatase [23]. During sporulation, Gip1 and Glc7 colocalize with septins throughout prospore membrane formation. Mutation of *GIP1 *leads to the failure of septins to localize to the prospore membrane, indicating that Gip1 may organize and form part of the septin complex.

Even though septin localization has been reported during spore formation [17], no data exist in regards to septin localization during the early phases of meiosis, from disassembly of the vegetative bud neck septin ring to the formation of the ring-like structures that surround the prospore membrane. Here, we provide evidence that the nutrient limitation signal that triggers the sporulation process also induces the disassembly of the septin bud neck ring and relocalization of the septin subunits to the nucleus. Septins remain associated with microtubules during the meiotic divisions, rather than being dispersed throughout the cytoplasm and later, during the spore morphogenesis program, they are detected associated to the nascent prospore membranes surrounding each nuclear lobe. Septin association to microtubules was also observed when cells were grown vegetatively in media containing non-fermentable carbon sources, such as acetate.

## Results

### Cdc11 localizes with microtubules during meiosis I and meiosis II

**Figure 1:**
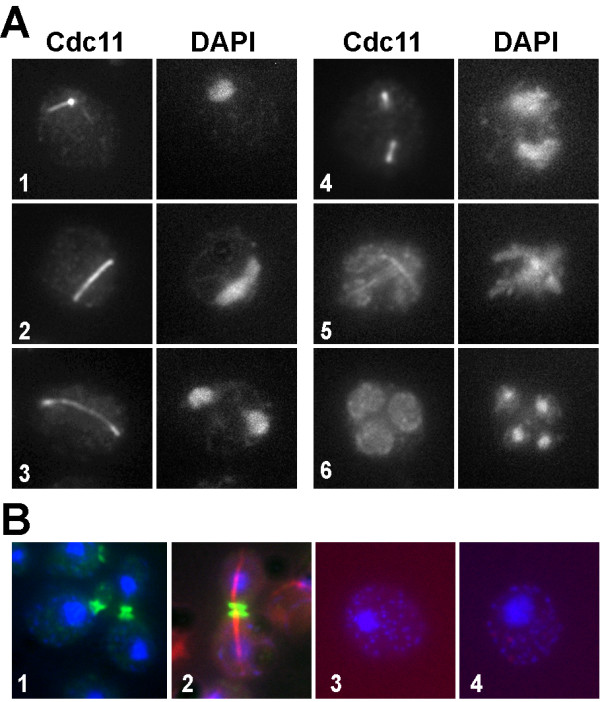
**Cdc11 localization during meiosis**. (A) Wild-type cells (AN120) incubated in sporulation medium were stained with anti-Cdc11 antibody and DAPI. Cells in different phases of meiosis are shown: (1), prophase; (2–3), anaphase I; (4), metaphase II; (5), anaphase II; (6), post-meiotic cell. (B) Exponentially growing wild-type cells were stained with anti-Cdc11 antibody (cell 1) or anti-Cdc11 and anti-tubulin antibodies (cell 2). Sporulating wild-type cells stained with anti-GFP and secondary anti-mouse Alexa 594 antibodies (cell 3) or the secondary antibodies anti-rabbit Alexa 488 and anti-mouse Alexa 633 (cell 4).

Previous studies reported that the Cdc11 septin localizes to the prospore membranes at the end of meiosis II [17]. Some fluorescence associated with the microtubules was also observed, although it was attributed to the strong tubulin signal. To analyze the localization of Cdc11 during the different phases of meiosis in greater detail, indirect immunofluorescence was used. The wild-type strain AN120 was incubated in sporulation medium and aliquots were collected at different times after the induction of sporulation. Septin localization was analyzed using anti-Cdc11 antibodies. Samples were stained with DAPI to monitor progression through meiosis. The results obtained indicated that Cdc11 clearly localized in a pattern that was reminiscent of the SPB and the astral microtubules during early meiosis. (Fig. 1A, cell 1). During later stages of meiosis, Cdc11 localized to spindle microtubules (Fig. 1A, cells 2–3) during meiosis and the prospore membrane (Fig. 1A, cells 4–5), as previously described [19]. Finally, in post-meiotic cells, Cdc11 appeared uniformly distributed throughout the membranes that surrounded the developing spore (Fig. 1A, cell 6).

**Figure 2:**
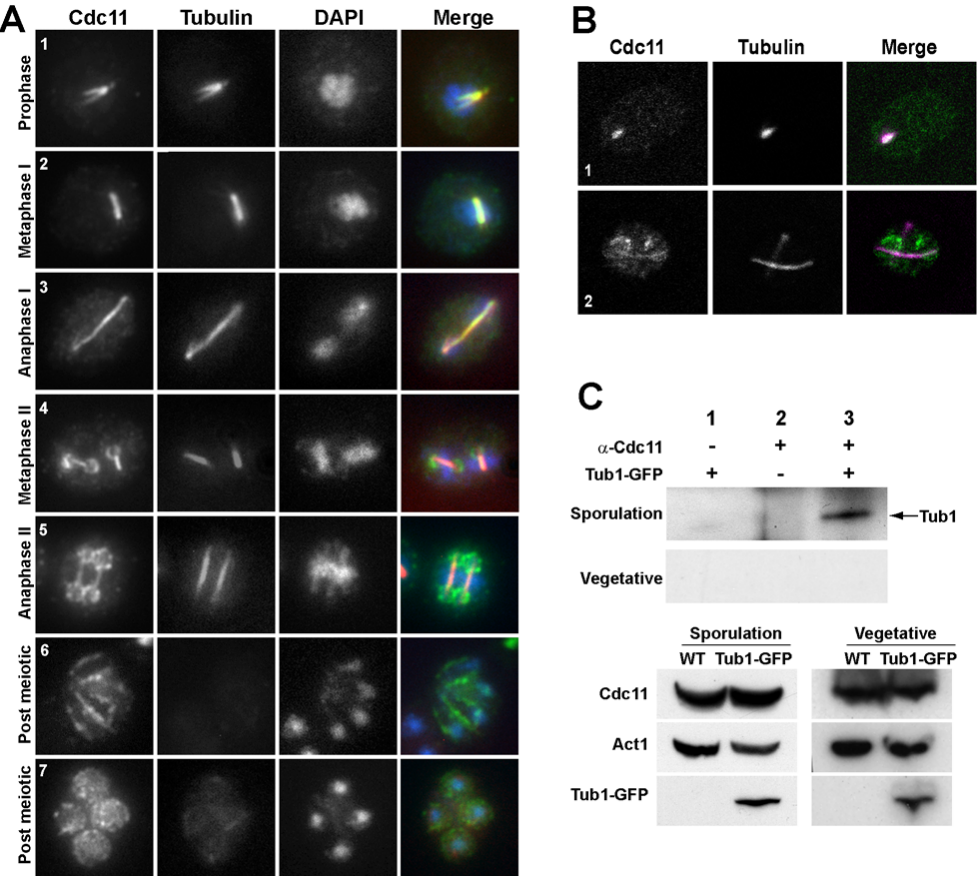
**Cdc11 localizes to tubulin cytoskeleton during meiosis before its association with the prospore membrane**. (A). Sporulating wild-type cells were stained with anti-Cdc11 and anti-tubulin antibodies and DAPI. Cells in different phases of meiosis are shown: (1), prophase; (2), metaphase I; (3) anaphase I; (4), metaphase II; (5), anaphase II; (6–7), post-meiotic cells. (B) Confocal microscopy of wild-type cells stained with anti-Cdc11 and anti-tubulin antibodies. The merged image shows Cdc11 (green) and tubulin (magenta) and white indicates areas where the two proteins colocalize. Cells in prophase (1) or anaphase II (2) are shown. (C) Cdc11 and Tub1 co-immunoprecipitate during sporulation. Protein extracts prepared from sporulating cells carrying TUB1-GFP (sporulation) or grown to mid-logarithmic phase (vegetative) were incubated in the presence (lane 3) or absence (lane 1) of anti-Cdc11 antibody. Extracts from the wild-type strain grown in the same conditions were used as controls (lane 2). Proteins were probed with anti-GFP antibody. The lower panels show a Western blot of the extracts used for the immunoprecipitations.

To confirm that Cdc11 localized to microtubules, we repeated the immunofluorescence using anti-Cdc11 and anti-tubulin antibodies. During meiosis I, Cdc11 was first observed in prophase cells in a structure that was coincident with the SPB and microtubules, as judged by the similar pattern observed for septins and microtubules (Fig. 2A, cell 1). During metaphase I and anaphase I, microtubules assembled the meiosis I spindle in the nucleus, and septin localization was also coincident (Fig. 2A, cells 2–3). As previously described [17], at the onset of meiosis II a fraction of Cdc11 was detected concentrated in ring-like structures that surrounded the four spindle-pole bodies (Fig. 2A, cell 4), which corresponded to the first stages of prospore membrane assembly, although some fluorescence was also associated with the meiotic spindle. In the later stages of meiosis II (Fig. 2A, cells 5–6), Cdc11 appeared localized around the prospore membrane as it expanded to engulf the nuclear lobes and also around the spores. In all cases, a fraction of Cdc11 remained associated with the microtubules during meiosis II until final disassembly of the spindle, at which time Cdc11 was only found in the membranes surrounding the spores (Fig. 2A, cell 7). Thus, Cdc11 co-localized with the tubulin cytoskeleton during meiosis I and meiosis II.

The specificity of the staining was assessed using several controls (Fig. 1B). Wild-type exponentially growing cells were stained with anti-Cdc11 antibody (Fig. 1B, cell 1) or doubly stained with anti-Cdc11 and anti-tubulin antibodies (Fig. 1B, cell 2). No Cdc11 signal was detected associated with microtubules or the mitotic spindle in any of the two controls. To further confirm the specificity of the staining, immunofluorescence was also performed in sporulating wild-type cells using anti-GFP antibodies and either of two secondary antibodies (anti-rabbit Alexa 488 and anti-mouse Alexa 633). No staining was detected in these controls (Fig. 1B, cells 3 and 4). Thus, these results rule out the possibility that the apparent Cdc11 localization to the microtubules might be due to mixed signals from different channels or non-specific antibody binding. They also confirm that Cdc11 is indeed associated with the spindle during greater part of meiosis.

To examine the localization of Cdc11 and microtubules more precisely, we used a spectral confocal microscope. The results showed that Cdc11 and tubulin had a similar localization through the different phases of meiosis (Fig. 2B). The co-localization between Cdc11 and tubulin after transfer to sporulation medium was observed in cells that had not initiated meiotic divisions (Fig. 2B, cell 1). Cdc11 localization with microtubules was maintained during meiosis I and meiosis II (cell 2). However, analysis of the overlaid signals indicated that there was a partial overlap in the localization of Cdc11 and tubulin, since there were some regions in which the signal was perfectly coincident, but also other regions in which only one of the signals was present. These results indicate that Cdc11 is associated with the microtubules and SPB during meiosis in *S. cerevisiae*, although the colocalization is not complete.

### Septins interact with microtubules during sporulation

To obtain physical evidence of the interaction between septins and microtubules during sporulation, immunoprecipitation assays were used. A wild-type strain expressing *TUB1-GFP *was transferred to sporulation medium and cells were collected after 10 h to prepare protein extracts. DAPI staining of the culture indicated that around 40% of the cells had 4 nuclei and around 5% were binucleated at this time point. Samples were immunoprecipitated using anti-Cdc11 antibody and the presence of tubulin in the immunoprecipitates was analyzed using anti-GFP antibody. The results indicated that the anti-Cdc11 antibody was able to immunoprecipitate Tub1-GFP from protein extracts prepared from sporulating cells (Fig. 2C, lane 3). The Cdc11-Tub1 interaction was specific, since no Tub1-GFP was immunoprecipitated from cells lacking Tub1-GFP (lane 2) or when anti-Cdc11 was omitted from the reaction (lane 1). It has been reported that microtubules interact with the septin ring during vegetative growth, and that this interaction is necessary for the alignment of the spindle along the mother-bud axis [24]. To test whether the Cdc11-Tub1 physical interaction was also detected during vegetative growth, we repeated the immunoprecipitation from exponentially growing vegetative cells and in this case, it was not possible to detect physical interaction between Tub1-GFP and Cdc11 (Fig. 2D). Thus, these results indicate that Cdc11 not only colocalizes with microtubules but that it is physically associated with tubulin during meiosis as well.

### Cdc10 and Spr28 also colocalize with microtubules during meiosis

**Figure 3:**
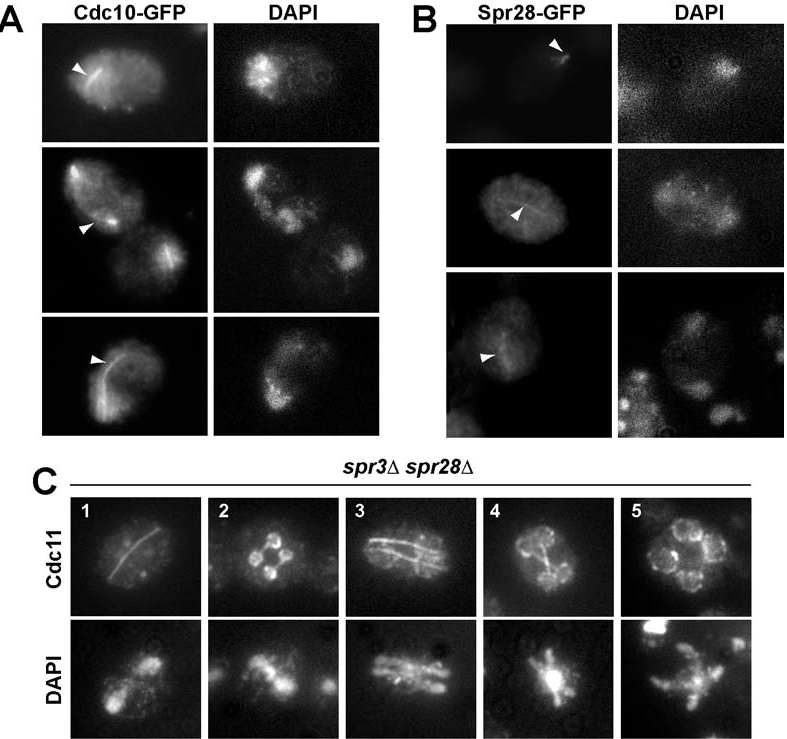
**Cdc10 and Spr28 also localize with microtubules**. (A) Wild-type cells carrying CDC10-GFP were stained with anti-GFP antibodies and DAPI. (B) Wild-type transformants carrying SPR28-GFP were stained with anti-GFP antibodies and DAPI. Arrowheads indicate Cdc10 (A) or Spr28 (B) localization to the microtubules. (C) Cdc11 localization in *spr3*Δ *spr28*Δ mutant. Sporulating cells (NY535) were stained with anti-Cdc11 antibody and DAPI. Cells in different phases of meiosis are shown: (1) anaphase I; (2), metaphase II; (3–4), anaphase II; (5), post-meiotic cells.

To test whether other septins had a similar distribution to that observed for Cdc11, we analyzed the localization of two additional septins: Cdc10 and the sporulation-specific septin Spr28. To this end, we used strains carrying either Cdc10-GFP or Spr28-GFP and the localization of the proteins was analyzed by indirect immunofluorescence using anti-GFP antibodies, since in our hands, the GFP fluorescence was very weak during meiosis I and could not be clearly distinguished from the cytoplasmic background. Cdc10 was also found associated with linear structures resembling meiotic spindles during meiosis I and meiosis II (Fig. 3A). Similarly, the sporulation-specific septin Spr28 also localized to microtubules (Fig. 3B). These results therefore suggest that other septins, not just Cdc11, associate with microtubules during meiosis in *S. cerevisiae*.

In vegetative cells, loss of one of the essential septins causes the disassembly of the septin ring at the bud neck. So, we decided to check if in the absence of some septins, such as the sporulation-specific septins Spr3 and Spr28, Cdc11 was able to localize correctly during sporulation. To test this, Cdc11 localization was analyzed by indirect immunofluorescence in a *spr3*Δ *spr28*Δ double mutant in sporulating conditions. The results showed that Cdc11 localization in the double mutant was generally similar to that in the wild-type strain (Fig. 3C), and it was found associated with meiotic spindles during meiosis I and meiosis II. Upon induction of prospore membrane assembly, Cdc11 localized to the nascent prospore membranes, although Cdc11 did not display the concentrated bar-like structures seen in wild type cells. These results indicate that the Cdc11 interaction with microtubules and its assembly in the structure that surrounds each nuclear lobe does not require the sporulation-specific septins Spr3 and Spr28 suggesting structural differences between septin complexes localized to different sites.

### *SPR3 *and *SPR28 *are required for septin organization during meiosis II

**Figure 4:**
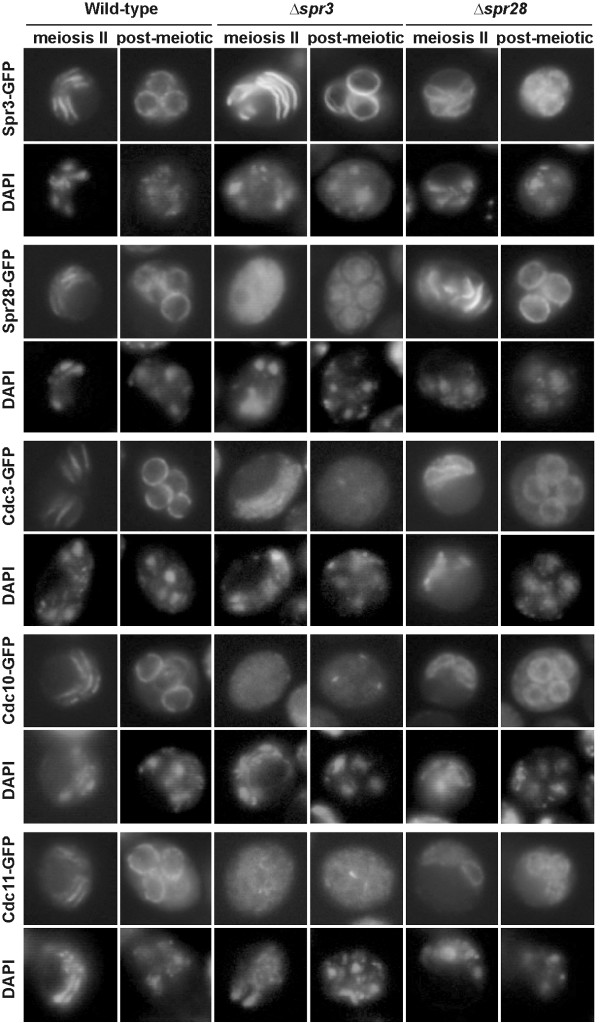
**Localization of septins in the prospore membrane of sporulating cells**. AN120 (wild-type), NY528 (*spr3*Δ) and NY703 (*spr28*Δ) strains were transformed with plasmids containing SPR3-GFP, SPR28-GFP, CDC3-GFP, CDC10-GFP or CDC11-GFP. Cells were sporulated, stained with DAPI and analyzed by fluorescence microscopy.

The failure of Cdc11 to localize to bars during meiosis II in the *spr3 spr28 *strain was reminiscent of the localization previously described for Cdc11 and Cdc3 in sporulating *spr3 *cells [17]. To investigate the requirements for septin organization in the prospore membrane during meiosis II further, the localizations of GFP fusions to five different septins -Cdc3, Cdc10, Cdc11, Spr3, and Spr28- were analyzed during sporulation in *spr3 *and *spr28 *single mutants. In wild type cells, all five proteins localized in the bar pattern during meiosis II and were distributed uniformly around the prospore membrane in post-meiotic cells (Fig. 4). In the *spr28 *single mutant, although the other four septins associated with the prospore membrane during meiosis II, they localized uniformly around the membrane rather than being restricted into bars. This indicates that Spr28 is necessary for the tight organization of septins on the prospore membrane. The phenotype of the *spr3 *mutant was even more severe. All four of the other septins are localized diffusely through the cytosol and failed to associate with the prospore membrane, though a weak association with the prospore membrane was sometimes seen, particularly for Cdc11. The delocalization of Cdc11-GFP visualized by GFP fluorescence is more dramatic than that seen using anti-Cdc11 antibodies (Fig. 3C). However, in all cases it is clear that the sporulation-specific septins are important for the proper association of Cdc11 with the prospore membrane during meiosis II.

### Cdc11 has different requirements for microtubule and prospore membrane localization

**Figure 5:**
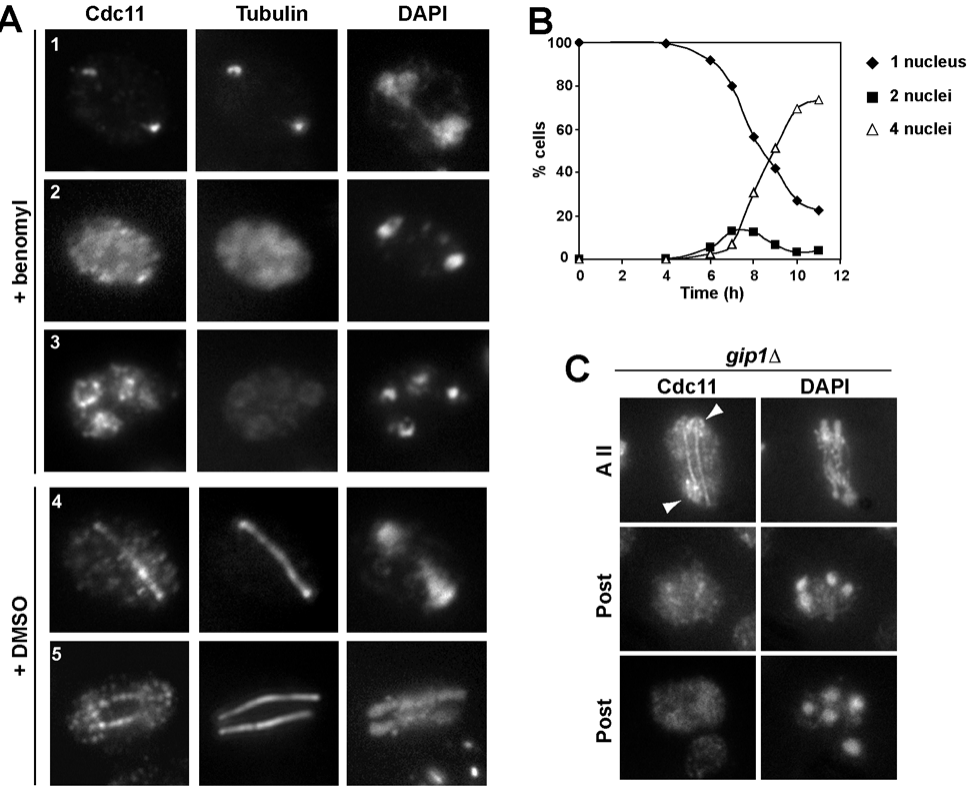
**Cdc11 localization to microtubule does not require Gip1**. (A) Sporulating wild-type cells were transferred to sporulation medium containing benomyl (120 μg/ml) (cells 1–3) or DMSO (cells 4–5) for 15 min and stained with anti-Cdc11 antibody and DAPI. Cells in different phases of meiosis are shown: (1–2), anaphase I; (3), anaphase II; (4), anaphase I; (5), anaphase II. (B) Progression through meiosis of cells in A. (C) *gip1*Δ mutants (NY501) were stained with anti-Cdc11 antibody and DAPI. Arrowheads indicate Cdc11 localized around the nascent prospore membranes. A II, anaphase II; post, post-meiotic cells.

To confirm that Cdc11 localization during meiosis was dependent on the microtubule cytoskeleton, cells were treated with the microtubule-depolymerizing drug benomyl. It has been reported that meiotic cells treated with benomyl arrest in G1 if microtubule perturbation occurs as they enter the meiotic cycle and in G2 if they are already undergoing the pre-meiotic S phase [25]. To completely depolymerize the microtubules without affecting progression through meiosis, cells were incubated in sporulation medium for 10 h and then transferred to sporulation medium containing 120 μg/ml benomyl. After 15 minutes of incubation with the drug, the cells were fixed and processed for immunofluorescence using anti-Cdc11 and anti-tubulin antibodies. Progression through meiosis was monitored by staining the cells with DAPI (Fig. 5B). Even though around 70% of the cells had completed meiosis II at this time point, cells in previous stages of meiosis were also present at lower frequency, and some examples are shown in figure 5A. Exposure to benomyl caused a complete depolymerization of microtubules (Fig. 5A, cells 1–3), and only the SPBs exhibited reactivity with anti-tubulin antibodies (cells 1–2). Interestingly, microtubule disorganization also resulted in Cdc11 delocalization from the linear structures found during early phases of meiosis, and this septin was only detected associated with the tubulin remaining in the SPBs (Fig. 5A, cell 1) or diffuse in the nucleus and cytoplasm (Fig. 5A, cell 2). However, in late meiosis II cells and post-meiotic cells, in which Cdc11 was found associated respectively with the microtubules and the prospore membrane or the prospore membrane only, microtubule depolymerization had no effects on Cdc11 localization on the prospore membrane (Fig. 5A, cell 3), suggesting that Cdc11 association with the prospore membrane is independent of microtubules and tubulin. In a mock-treated culture, no effects on microtubule or Cdc11 localization were observed (Fig. 5A, cells 4–5). Therefore, these results indicate that Cdc11 localization to the meiotic spindle requires the stability of the microtubule cytoskeleton.

Gip1 is a developmentally regulated protein phosphatase 1-interacting protein that targets the Glc7 protein phosphatase to the growing prospore membranes. This Gip1-Glc7 complex is required for proper septin localization around the prospore membranes [23]. To test whether Gip1 was also required for the septin interaction with microtubules and meiotic spindles, Cdc11 localization was analyzed in a *gip1*Δ mutant. In this strain, we found that Cdc11 was able to bind microtubules and that it localized to the meiotic spindles during all phases of meiosis, similar to wild-type cells (Fig. 5C). Prospore membrane localization was seen in only a small fraction of cells (less than 20%), showing a ring-like pattern surrounding four nuclei, as noted previously for the septins Spr3 and Spr28 in *gip1 *mutants [23]. The appearance of this small fraction of cells suggests that *GIP1 *is not required for the dissociation of Cdc11 from the microtubules or for its nuclear export. That is, Gip1 is required for proper septin organization around the prospore membrane, but is not required for the septin interaction with microtubules and meiotic spindles during meiosis. Thus, the Cdc11-microtubule interaction is differently regulated from that of the Cdc11-PSM interaction.

### Temporal pattern of septin localization to microtubules and prospore membranes

**Figure 6:**
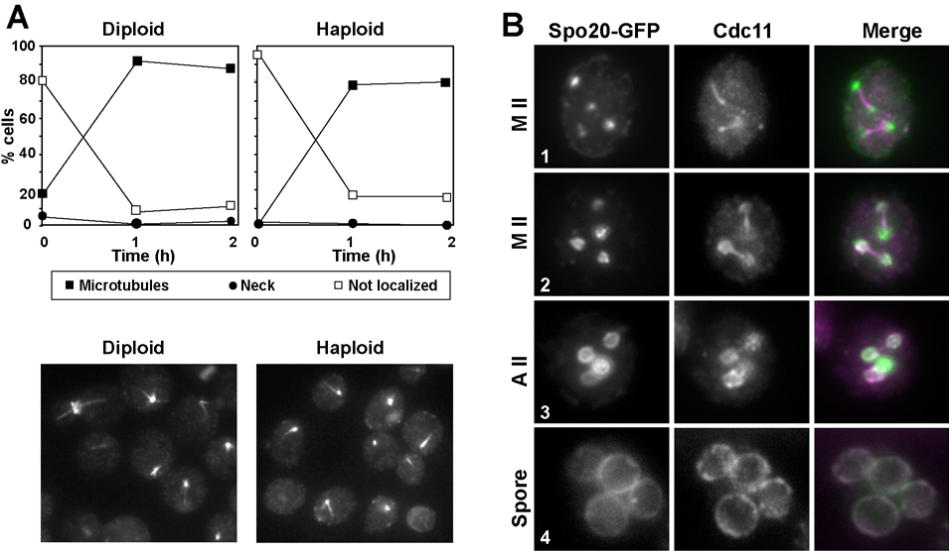
**Temporal pattern of Cdc11 localization to microtubules**. (A) AN120 diploid cells (left panel) or AN117-16D haploid cells (right panel) were grown in YPAcetate medium before transferring to sporulation medium. Aliquots were then taken at 0, 1 and 2 h and stained with anti-Cdc11 antibody. The graph shows the percentage of cells with different Cdc11 stainings: bud neck (black circles), microtubules (black squares) or not localized (white squares). At least 200 cells were counted. (B) Wild-type cells (AN120) carrying *GFP-SPO20*^51–91 ^(plasmid pG20) were sporulated and stained with anti-Cdc11 and DAPI. Images of Spo20-GFP, Cdc11 and DAPI are shown. The merged image shows Spo20 (green) and Cdc11 (magenta). Colocalization is indicated in white. Metaphase II (1–2), anaphase II (3), or post-meiotic (4) cells are shown.

We next analyzed the timing of septin localization to microtubules and from the microtubules to the PSM during meiosis in more detail. Cells were grown to stationary phase in YPAcetate before transfer to the sporulation medium. Samples were then taken at short intervals after transfer to sporulation medium and stained with anti-Cdc11 antibody. At time 0 h, most of the cells were unbudded and around 80% of them showed no specific Cdc11 staining (Fig. 6A, left panel). However, after 1 h of incubation in sporulation medium, Cdc11 was found to be associated with microtubules in more than 90% of the cells. These results therefore suggest that septins localize to microtubules shortly after transfer to sporulation medium, before the onset of the sporulation program.

To analyze the moment at which septins move from the meiosis II spindle to the nascent prospore membrane in more detail, Cdc11 localization was analyzed in cells carrying *GFP*-*SPO20*^51–91^. Spo20 is a sporulation-specific protein that is required for spore morphogenesis [7]. Spo20 is necessary for the coalescence of precursor vesicles on the SPB to form the prospore membrane, and it localizes to this nascent structure during metaphase II [26]. Colocalization experiments using GFP-Spo20^51–91 ^and anti-Cdc11 revealed that during metaphase II, Spo20 started to accumulate around the SPB and nascent prospore membranes, and Spo20 localization to this region was usually detected before Cdc11 appearance at the PSMs (Fig. 6B, cell 1). Cdc11 started to assemble the ring-like structures that colocalized with Spo20 in metaphase II (Fig. 6B, cell 2), as previously described [17]. As the prospore membrane grew in size during anaphase II, Cdc11 signal increased at the prospore membrane (Fig. 6B, cell 3). The association with the prospore membrane was then maintained in post-meiotic cells during spore development (Fig. 6B, cell 4). These results suggest that the signal that induces Cdc11 localization to the nascent prospore membranes is dependent on the initiation of the differentiation program and occurs after prospore membrane assembly has started.

### Septin association to microtubules is dependent on the nutrient starvation signal

When entering sporulation, diploid cells are exposed to two different nutritional stimuli, exposure to a non-fermentable carbon source and nitrogen deprivation. As a consequence of these signals, diploid cells initiate a developmental program that promotes the formation of spores. To test whether bud neck septin ring disassembly and relocalization of the septin subunits to the microtubules was a consequence of the induction of the meiotic program or a response to the nutrient starvation signal, Cdc11 localization was analyzed in the AN117-16D strain, one of the parental haploid strains of AN120. Haploid cells were incubated for 24 h in YPAcetate medium before transfer to the sporulation medium, samples were removed at different time-points and stained with anti-Cdc11 antibody to analyze Cdc11 localization. The results indicated that Cdc11 associates to microtubules in haploid cells with similar kinetics to that previously described for diploid cells (Fig. 6A, right panel). Thus, these data indicate that septin relocalization to microtubules occurs shortly after transfer to sporulation medium, and that nutrient limitation, and not the induction of the sporulation program, must be the signal that triggers this reorganization of the septins.

**Figure 7:**
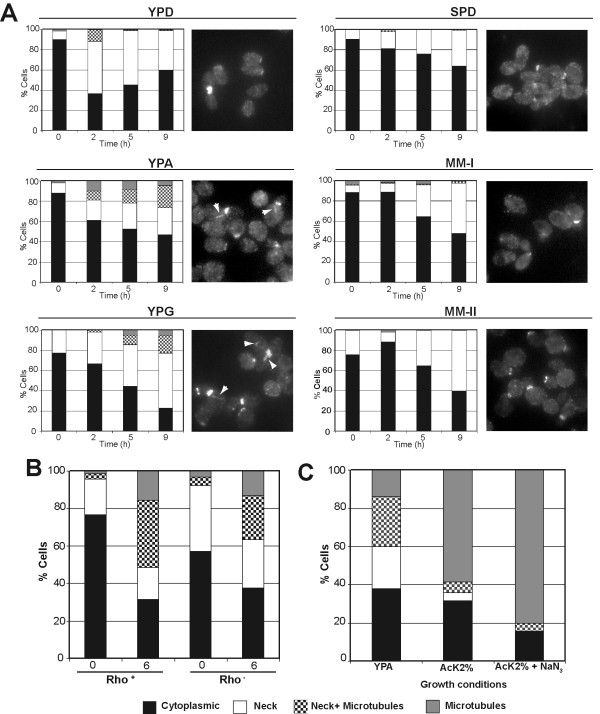
**Non-fermentable carbon sources induce Cdc11 localization to microtubules**. (A) AN117-16D haploid cells were inoculated in media containing glucose (YPD), acetate (YPA) or glycerol (YPG) as carbon sources or in media with poor nitrogen sources (SPD or MM-I). For MM-II medium, AN999 (*MATα, lys1*) was used. Cells were stained with anti-Cdc11 antibodies. The percentage of cells with bud neck (white), bud neck and microtubules (squares), microtubules (grey) or diffuse cytoplasmic Cdc11 staining (black) is shown in the graphs. Representative cells are also shown. (B) Cdc11 distribution in AN117-16D haploid cells or the *rho*^- ^derivative grown in YPA. (C) AN117-16D haploid cells were grown in YPA for 16h and then transferred to AcK 2% or AcK 2% containing 15 mM sodium azide. The percentage of cells with different Cdc11 localizations 4 h after the inoculation is shown in the graph.

In the course of these experiments, we noticed that some Cdc11 was present at the microtubules in some cells even at time 0 (cells growing in YPAcetate), suggesting that this septin could also interact with the tubulin cytoskeleton during vegetative growth depending on nutritional growth conditions. So, to analyze in further detail the nature of the nutrient signal, we studied Cdc11 localization in haploid cells that were vegetatively grown in different media, collecting samples at different stages of the growth curve. We used media containing different carbon sources (such as YPD, YPAcetate and YPGlycerol) and media containing poor nitrogen sources (see Materials and Methods) to distinguish different nutritional stimuli. Our results showed that nitrogen starvation did not alter the distribution of Cdc11 compared with cells grown in YPD (Fig. 7A). However, growth in non-fermentable carbon sources, such as acetate and glycerol, resulted in an increase in the percentage of cells displaying microtubule staining or a mixed pattern, both the neck and microtubules (Fig. 7A). Other carbon sources, such as glucose or raffinose, did not modify septin localization at the bud neck (Fig. 7A and data not shown). Thus, these data suggest that Cdc11 translocation from the neck to the microtubules depends on the nutritional signal, primarily on the carbon source, and this response to the carbon source is enhanced by nitrogen starvation.

One of the consequences of the growth in the presence of non-fermentable carbon sources, such as acetate, is a change in the metabolism from fermentation to respiration. To analyze whether septin association to microtubules was a consequence of this metabolic change, a *rho*^- ^strain, lacking mitochondria and being unable to respirate, was isolated from the haploid AN117-16D. Both strains (*rho*^+ ^and *rho*^-^) were grown in YPD overnight and the cells were then inoculated to YPAcetate to analyze Cdc11 localization when cells cannot respirate. The results indicated that Cdc11 distribution (neck or microtubules) was similar in the two strains (Fig. 7B). To confirm this result, the haploid strain AN117-16D (*rho*^+^) was grown in YPAcetate to mid-logarithmic phase and transferred to AcK 2% (the strongest inducer of Cdc11 association to microtubules observed) with or without sodium azide to inhibit respiration and Cdc11 distribution under these conditions was analyzed. Samples were taken at different times after the inoculation and stained with anti-Cdc11 antibodies. No significant differences were observed between the two conditions, (Fig.7C) suggesting that inhibition of the electron transfer chain does not interfere with septin localization to microtubules. Together, these results indicate that septin relocalization to microtubules is not a consequence of the induction of the sporulation program, but it is specifically induced by a nutritional signal depending on the carbon source. Furthermore, the metabolic change from fermentation to respiration does not promote septin association with microtubules.

## Discussion

Septins are conserved GTP-binding proteins that play essential roles in morphogenesis in fungi and higher eukaryotic cells. In budding yeast, septins form a series of filaments that assemble as a ring at the bud neck during vegetative growth. Septin rings are thought to function as a scaffold to recruit proteins to the bud neck and as a boundary domain to restrict diffusion during budding and cytokinesis (reviewed in [10-12]). Previous reports have shown that in *S. cerevisiae *septins localize to the PSM during sporogenesis, although it is not known what their function is in this process. It has been proposed that septins could be required for the correct assembly of PSMs, although mutants lacking several septins have no defects in spore formation, which suggests that septins could act redundantly during this process [2]. In the course of the analysis of the role of the Cdc15 kinase during sporulation [27], we used Cdc11 as a marker for analysis of PSM morphology by indirect immunofluorescence and noticed that Cdc11 had a localization pattern similar to that of the microtubule cytoskeleton at early time-points of the sporulation time-course. In addition, previous reports had already observed the localization of several septins, such as Cdc11 or Spr3, at the microtubules although it was assumed that it was a cross of fluorescence channels [17,28].

### Dynamic behavior of septins during sporulation

In *S. cerevisiae*, septin localization at the bud neck during vegetative growth is relatively constant [9,29]. By contrast, our results show here that septins localization and behavior is much more variable through the process of sporulation. Analysis of septin localization during sporulation has been addressed by using two different techniques, *in vivo *analysis using GFP fusions and immunofluorescence in fixed cells. Microtubule association of Cdc11 and other septins was mainly detected by indirect immunofluorescence, while it was not detected using GFP fusions. Conversely, prospore membrane localization was clearly seen with both techniques. One possible reason to explain these differences is the relative amounts of septins associated with these two structures. It could be that the fraction of septins that interact with the microtubule cytoskeleton is low and that its interaction is very dynamic, making difficult its detection *in vivo*. On the other hand, fixation of the cells for immunofluorescence could stabilize the fraction of septins associated to microtubules or modify the proteins, facilitating its detection with specific antibodies.

During sporulation, Cdc11 localizes to different cell compartments; the SPBs, astral microtubules (cytoplasm), the meiotic spindle (nucleus) and the PSM. The association of Cdc11 with microtubules was confirmed by immunoprecipitation experiments, although our results do not indicate whether the interaction between tubulin and septins is direct or mediated by other proteins. Furthermore, analysis of Cdc10 and Spr28 localization indicated that septin-microtubules interaction during sporulation is not exclusive to Cdc11, but it is a characteristic of other septins as well. Spr28 and Cdc10 localization to the microtubules was weak but both proteins strongly accumulated at the PSMs later in sporogenesis, whereas the Cdc11 signal was more intense in the microtubule cytoskeleton and declined during spore wall formation. Colocalization of Spr28-GFP and Cdc11 (data not shown) supported the idea that affinity for microtubules or the PSM is different. Although this observation should be verified in greater detail, it is possible that these differences only reflect the different temporal pattern of expression of the genes during sporulation, since *SPR28 *and *CDC10 *are induced as middle sporulation genes while *CDC11 *is constitutively expressed, but at a lower level [20].

Our experiments show that tubulin-Cdc11 interaction and Cdc11-PSM interaction during sporulation are independent. This is supported by several experiments: First, depolymerization of the microtubule cytoskeleton using benomyl resulted in delocalization of the fraction of Cdc11 that was associated to tubulin, but it had no effect on those molecules that were localized to the PSM. In contrast, the absence of *GIP1 *is sufficient to disorganize septins at the PSM [23], but has no effects on the septins localizing to the microtubule cytoskeleton. Second, nutritional experiments and the fact that Spo20 vesicles arrived earlier than Cdc11 to the meiotic outer plate of the SPB suggests that Cdc11 localization to microtubules and PSM are independently regulated and respond to different signals. Thus, our results clearly indicate that Cdc11-microtubule association is triggered by a nutritional signal and also occurs during vegetative growth, while septin-PSM association is part of the developmental program that leads to the production of spores [17,19,23].

The genetic requirements for these different localizations are also distinct during sporulation. During vegetative growth, absence of an essential septin results in defects in the localization of the other septins and septin ring disassembly. In contrast, during sporulation, Cdc11 can localize to microtubules in the absence of *SPR3 *and *SPR28 *or *GIP1*, but its subsequent localization on the prospore membrane is altered. In the absence of *SPR28*, the remaining septins cannot form discrete bars but are localized uniformly around the prospore membrane throughout meiosis II. Mutation of *SPR3 *is distinct from *spr28*Δ in that not just the sheets, but the association of the remaining septins with the prospore membrane is greatly reduced. In this respect, mutation of *SPR3 *resembles a *gip1 *mutant [23]. Thus, though the function of the septins at the prospore membrane remains obscure, the sporulation-specific septins play direct and distinct roles in the organization of septin complexes during sporulation. The differential genetic requirements for septin localization at different stages of sporulation suggest that the organization of the septins themselves is changing during this process. It is noteworthy that the ability of Spr3 to associate with other septins depends on the phase of the life cycle. When ectopically expressed in vegetative cells, Spr3 does not localize with other septins at the bud neck [17]. Taken together, these observations suggest that both the localization and the organization of the septins are changing dynamically throughout sporulation.

One possible explanation for these observations is that the stoichiometry of the septin subunits could be different during the successive phases of the sporulation process, and these differences could be important for their association to microtubules or PSM. Structural studies of mammalian septins suggest that the basic organization of a septin filament is a heterotrimer [30]. It may be that different combinations of septins produce filaments with different properties. If so, understanding what these changes are and how they are regulated in yeast could prove insightful in other organisms which, like *S. cerevisiae*, express more than three septin proteins.

### Septin-Tubulin interactions

Septin-tubulin interactions had been previously observed in budding yeast [28], but this interaction was not analyzed in this organism. However, in mammalian cells, septins localize not only to the plasma membrane but also throughout the cytoplasm, interacting with the microtubule and actin cytoskeletons, where they act as a scaffold for cytoskeleton-binding proteins (reviewed in [15]). For example, the mammalian septin Sept9 (formerly known as MSF) colocalizes during interphase with actin, microtubules and another mammalian septin, Sept2 (Nedd5) during mitosis [31]. Sept2 filaments are associated with actin fibers, and play a role in stabilization of actin stress fibers preventing actin turnover [32-34]. It has also been shown that Sept2 and Sept9 can align along microtubules during interphase in PC12 and HeLa cells [35,36], and Sept2 has been reported to associate with spindle microtubules during mitosis [37]. Furthermore, in mammalian cells septins not only interact with microtubules or actin cytoskeleton, but also control their organization and stability. This is the case of the mammalian septin complex composed of Sept2/6/7, which regulates microtubule stability through an interaction with the microtubule-binding protein MAP4 [38] or actin organization through nuclear accumulation of the adaptor protein NCK [34]. Moreover, it has also been suggested that Sept9 might have a function in microtubule organization during cytokinesis [31]. In yeast, it is unlikely that septins may play a similar role in microtubule cytoskeleton organization, although it has been reported that microtubules interact with the septin ring to align the mitotic spindle along the mother-bud axis during vegetative growth [24].

### Septin localization to microtubules and nutritional stimuli

In unicellular organisms, sporulation is a process necessary for cells to survive when environmental conditions change. In *S. cerevisiae*, sporulation is triggered in response to nitrogen deprivation and, this process is induced when cells are grown in a non-fermentable carbon source such as acetate. Analysis of Cdc11 localization in sporulating cells indicated that Cdc11 localized to the microtubules shortly after transfer to sporulation medium, and the same pattern was observed also in haploid cells, suggesting that the nutritional stimulus is the main signal responsible for the change in septin distribution. A deeper analysis of Cdc11 localization to microtubules in haploid cells has resulted in the following conclusions: 1) Cdc11 associates with microtubules during vegetative growth under certain nutritional stimuli, so Cdc11 can interact with tubulin independently of the developmental stage; 2) Non-fermentable carbon sources, such as acetate or glycerol, were the signal triggering Cdc11 association with microtubules; 3) the presence of non-fermentable carbon sources *per se *and not the transition to the respiratory metabolism is the origin of the stimulus that promotes Cdc11 localization to the tubulin cytoskeleton; and 4) though nitrogen starvation cannot trigger septin relocalization to microtubules, the absence of nitrogen enhances the effect of a non-fermentable carbon source. Thus, these results suggest that Cdc11 has the ability to interact to tubulin at different moments of the yeast life cycle.

At the moment, we do not know the exact nature of the signal that triggers Cdc11 association to microtubules. One possibility is that it involves changes in the intracellular concentration of a secondary messenger, such as cAMP or intracellular pH, allowing the interaction of tubulin and septins, two families of structural proteins, to maintain cytoskeleton polymerization under these conditions. Whether these complexes constitute only a response to intracellular changes or they have an active role has yet to be elucidated. The first possibility in good agreement with results from mammalian cells, in which Sept2 polymerizes forming structures inside the cytoplasm without any defined function when actin cytoskeleton is perturbed [32]. In budding yeast, release of septins from the neck and polymerization of proteins in the cytoplasm could be a mechanism to store proteins temporarily until intracellular conditions would be reestablished to promote an active role of these proteins.

## Conclusion

Nutrient limitation in *S. cerevisiae *triggers the sporulation process, but it also induces the disassembly of the septin bud neck ring and relocalization of the septin subunits to the nucleus. Septins remain associated with microtubules during the meiotic divisions and later, during spore morphogenesis, they are detected associated to the nascent prospore membranes surrounding each nuclear lobe. Septin association to microtubules also occurs during growth in non-fermentable carbon sources.

## Methods

### Strains, growth conditions and plasmids

The wild-type diploid strain AN120 (*MAT*a/*MAT*α *ARG4/arg4-NspI his3Δ SK/his3Δ SK hoΔ ::LYS2/hoΔ ::LYS2 leu2/leu2 lys2/lys2 RME1/rme1Δ::LEU2 trp1::hisG/trp1::hisG ura3/ura3*), its parental haploid strains AN117-16D (*MATa ura3 leu2 trp1::hisG his3Δ SK lys2 hoΔ ::LYS2*) and AN117-4B (*MAT*α *arg4-NspI his3Δ SK hoΔ ::LYS2 ura3 leu2 lys2 rme1Δ::LEU2 trp1::hisG*) used in this study are derived from the fast-sporulating SK1 background. Strain NY535 (*MAT*a/*MAT*α*his3/his3 ura3/ura3 leu2/leu2 lys2/lys2 trp1::hisG/trp1::hisG arg4-NspI/ARG4 ho::LYS2/ho::LYS2 RME1/rme1::LEU2 spr28::KanMX6/spr28::KanMX6 spr3::TRP1/spr3::TRP1*) contains a deletion of the two sporulation-specific septins, *SPR3 *and *SPR28*. Strains NY528 and NY703 were constructed by PCR mediated replacement of *SPR3 *and *SPR28*, respectively, with the *S. pombe his5*^+ ^gene in the haploids AN117-16D and AN117-4B followed by mating of the resulting haploids to obtain homozygous deletion diploids. SEP38 is isogenic to AN120, carrying the *TUB1-GFP *fusion integrated at the *TRP1 *locus. Strain NY501 (*MATa/MATα his3/his3 ura3/ura3 trp1::hisG/trp1::hisG leu2/leu2 arg4-NspI/ARG4 lys2/lys2 ho::LYS2/ho::LYS2 rme1::LEU2/RME1 gip1::HIS3/gip1::HIS3*) has been described previously [23]. AN117-16D *rho*^- ^is a respiratory deficient derivative of AN117-16D. It was obtained by growing cells in YPD plates for 24 h and then selecting clones unable to grow in medium containing glycerol as carbon source.

For sporulation, overnight liquid cultures were grown in YPD or SC media [39] to saturation. Cultures were then diluted 1:300 in YPAcetate medium (1% yeast extract, 2% peptone, 2% potassium acetate), grown to midlog phase, and transferred to 2% potassium acetate at a final concentration of 3 × 10^7 ^cells/ml [7]. Aliquots of the cultures were removed at different times. Yeast strains were transformed with the lithium acetate protocol [40]. For nutritional experiments, media containing different carbon sources were used: YPD (2% glucose), YPA (2% acetate), YPG (3% glycerol), SPD medium supplemented with the required amino acids [41]. MM-I is a nitrogen-limited medium (Ammonium sulfate 7.5 mM, glucose 330 mM)[42] while MM-II is a defined medium poor in nitrogen (glucose 2%, proline 1 g/l and lysine 1 g/l).

Plasmid pG20 contains *GFP-SPO20*^51–91 ^cloned in pRS424 [8] and pSB7 contains *SPR28-GFP *cloned in pRS314 [19]. pBS237 contains *TUB1-GFP *and was kindly provided by Dr. Pedro San Segundo (Instituto Microbiología Bioquímica, Spain). To integrate it, the plasmid was linearized by digestion with *Hin*dIII before transformation. To construct pSB203, *SPR3 *was first *GFP *tagged in the genome using PCR-mediated integration. The *SPR3-GFP *fusion gene was then amplified from genomic DNA and cloned into the polylinker of pRS314 as an *Xho*I-*Eco*RI fragment. Plasmid pSB211 expressing *CDC11-GFP *was similarly constructed by amplification of the fusion gene from genomic DNA and subsequent cloning into pRS314 as an *Xho*I-*Sma*I fragment. pM2 (*CDC10-GFP*) and pRS316-*CDC3-GFP *have been described elsewhere [43,44].

### Microscopy techniques

Indirect immunofluorescence was performed as previously described [45]. Primary antibodies were diluted 1:100 and incubated for 1 h. Samples were washed 5 times with PBS-BSA 1% and incubated with secondary antibodies (1:200) for 1 h. After incubation, antibodies were removed by washing thoroughly with PBS buffer. The primary antibodies used were: anti-tubulin (Sigma 75168); anti-Cdc11 (sc7170, Santa Cruz Biotechnology) or anti-GFP (Living colors 8371, Becton Dickinson). Different secondary antibodies coupled to Alexa Fluor 488, 594 or 633 (Invitrogen Molecular Probes) were used. Samples were mounted in mounting media (Vectashield mounting medium; Vector Laboratories, CA) and observed under a Leica DMXRA microscope equipped for Nomarski optics and epifluorescence. Images were captured using a Photometrics Sensys CCD camera. Images shown correspond to a single focal plane. Confocal microscopy was performed on a Leica TCS SL spectral confocal microscope and the images were analyzed with the Leica Confocal Software.

For microtubule depolymerization, AN120 cells were sporulated for 10 h and then resuspended in 2% potassium acetate plus 120 μg/ml benomyl, prepared as described elsewhere [25]. Cells were then fixed overnight in 4% formaldehyde at 4°C and stained with anti-Cdc11 and anti-tubulin antibodies.

### Immunoprecipitation

Protein extracts and immunoprecipitations were prepared as described [46]. Samples were separated in NUPAGE Novex 10% Bis-Tris gels (Invitrogen) according to recommendations of the supplier. Proteins were transferred to P-Hybond membranes (GE Healthcare) using an X-Cell Sure Lock Mini-Cell system (Invitrogen). Anti-actin (69100, ICN), anti-Cdc11 (Y415 sc7170, Santa Cruz Biotechnology) or anti-GFP (Living colors 8371, Becton Dickinson) were used as primary antibodies. Horseradish peroxidase-conjugated anti-mouse antibody (NA931V, GE Healthcare) or anti-rabbit antibody (NA934, GE Healthcare) were used as secondary antibodies. Detection of proteins was performed using the ECL kit (GE Healthcare).

## Authors' contributions

MEP–H performed most of the experiments, participated in their design and helped to draft the manuscript. YA–P carried out most of the indirect immunofluorescences performed in this study. HT was involved in septin localization *in vivo*. AMN participated in the design of the study and participated in writing of the manuscript. FdR participated in the design and helped to draft the manuscript. CRVdA was the project leader, participated in the design of experiment and prepared the manuscript. All authors read and approved the final manuscript.
